# Effect of Ion Irradiation on Corrosion Behavior of Two Medium-Entropy Alloy Coatings in Simulated PWR Primary Water

**DOI:** 10.3390/ma19153319

**Published:** 2026-08-04

**Authors:** Hongyang Xin, Changfeng Dong, Jianjun Mao, Tao Peng, Wei Zhang, Zhien Ning, Xiaoyong Wu

**Affiliations:** 1 The First Sub-Institute, Nuclear Power Institute of China, Chengdu 610041, China; dcf@npic.ac.cn (C.D.); mao0707@npic.ac.cn (J.M.); pengtaoustb@163.com (T.P.); 2190016@stu.neu.edu.cn (W.Z.); np201006@163.com (Z.N.); 2 The Fourth Sub-Institute, Nuclear Power Institute of China, Chengdu 610041, China

**Keywords:** medium-entropy alloy coating, ion irradiation, high-temperature high pressure water corrosion

## Abstract

We investigated the impact of Au ion irradiation on the corrosion behavior of medium-entropy alloy coatings (MEAs), with and without Al addition, in simulated primary water of pressurized water reactors (PWRs). The results indicated the formation of double-layer oxide films on the surfaces of both types of coatings, consisting of outer oxide particles and a protective inner oxide layer. The high-fluence irradiation modified the microstructures of both coatings and accelerated their corrosion kinetics. However, the coating with a slight addition of Al demonstrated superior post-irradiation corrosion resistance owing to its enhanced lattice distortion effect within the system, inhibited atomic diffusion, and reduced impact of irradiation on its phase structure. A comprehensive discussion was carried out on the corrosion processes of the coatings, both irradiated and non-irradiated.

## 1. Introduction

Compared to the medium- and long-term objectives in developing accident tolerant fuel (ATF) cladding for pressurized water reactors, the application of protective coatings on the surface of existing zirconium alloys represents a short-term goal that is more readily achievable and applicable. The methodologies for coated zirconium alloy cladding are compatible with the established and mature zirconium alloy cladding technology systems, and can effectively reduce both the heat generation rate and the total amount of heat produced during a loss-of-coolant accident and beyond design basis accident [1,2].

Considering the service requirements of pressurized water reactors (PWRs) under both normal and accident conditions, the effective coating applied to the surface of zirconium alloy should exhibit the following characteristics and properties: (a) Good physical and chemical stability under multi-field coupling and in the event of high-temperature steam oxidation accidents; (b) Excellent corrosion resistance, capable of forming a stable oxide layer to prevent reactions between the oxidant and the substrate; (c) A low neutron absorption cross-section, resulting in minimal changes to reactor core physics; (d) Excellent radiation resistance, avoiding the inclusion of elements that can undergo nuclear transmutation reactions with neutrons; (e) Outstanding adhesion between the coating and substrate, along with thermal shock resistance [3].

Within the radiation field of the reactor core, ATF cladding coating materials are exposed to harsh operational environments characterized by intense neutron radiation, corrosive media, high pressure, temperature gradients, and elevated stresses. The high-energy particles generated by nuclear reactions interact vigorously with the coating materials, disrupting their atomic structure and leading to the formation of a substantial number of supersaturated point defects and defect clusters. These defect clusters, induced by irradiation, aggregate and migrate, thereby enhancing elemental diffusion, altering chemical composition and phase structure, and inducing grain boundary segregation. This process accelerates the degradation of irradiated materials, particularly under conditions of high-temperature and high-pressure water coolant corrosion. Consequently, investigating the intrinsic relationship between the microstructural evolution of irradiated structural materials and the corresponding variations in mechanical and corrosion resistance is of paramount importance for the design of irradiation-resistant PWR fuel cladding coatings [4,5].

To date, extensive research has been conducted on several types of candidate coating materials for ATF cladding, encompassing ceramic coatings (such as SiC, CrSi, Ti_3_SiC_2_, and TiAlN), metallic coatings (including Cr, CrNb, FeCrAl, and high-entropy alloys), and multilayer coatings (for instance, TiN/TiAlN, Mo/FeCrAl, and Cr–CrN/TiSiN–Cr) [6,7,8,9]. Among these, Cr and FeCrAl coatings have undergone thorough evaluation for their resistance to high-temperature steam oxidation and irradiation tolerance [6,7,10]. Meanwhile, high- and medium-entropy alloy coatings have garnered increasing attention owing to their pronounced lattice distortion and sluggish diffusion effect, which contribute favorably to irradiation resistance and corrosion stability [11,12]. Under the individual research conditions of irradiation damage effect assessment, high-temperature pressurized water corrosion testing, and high-temperature steam oxidation experiments, these coatings have demonstrated exceptional performance. Nevertheless, the actual operational conditions of reactors involve complexly coupled fields, necessitating further investigation into the behavior of coating materials under such multi-field coupling scenarios. Consequently, the design of innovative candidate ATF coating materials capable of withstanding exceedingly harsh service environments remains a pivotal task [13].

Recently, medium-entropy alloy (MEA) coatings have garnered significant attention due to their admirable irradiation resistance, high strength, hardness, excellent corrosion resistance, and high phase structural stability. According to the literature, MEA coatings prepared by magnetron sputtering tend to be dominated by amorphous structures. This is likely due to the combined effect of the high cooling rate inherent in magnetron sputtering and the suppression of grain growth by high mixing entropy under non-thermodynamic equilibrium conditions. The design of such amorphous coatings offers distinct advantages: (1) The long-range disordered structure of medium-entropy amorphous coatings makes them more suitable for irradiated environments compared to conventional crystalline materials, as they can absorb more radiation-induced damage and reduce the formation of conventional crystalline defects [14]; (2) Amorphous coatings offer superior corrosion resistance compared to traditional crystalline materials of the same composition, owing to their microstructural and compositional homogeneity and the absence of crystal defects such as grain boundaries and second-phase particles [15]. Therefore, amorphous MEA coatings represent a promising candidate material for application in the harsh service environment of PWRs, warranting a detailed evaluation of their comprehensive properties [16].

Under intense neutron irradiation, the coolant can expedite the corrosion of reactor materials, thereby compromising their structural integrity [17]. Consequently, investigating the synergistic effects of irradiation and corrosion is of paramount importance. Given the experimental constraints, researchers often resort to ion irradiation followed by corrosion testing as a viable alternative. Zhang et al. explored the impact of ion irradiation on the corrosion behavior of two high-entropy alloys (Cr_0.25_Mo_0.5_NbTiV and Mo_0.5_NbTiV_0.5_Zr_0.25_) in high-temperature pressurized water [18]. The findings indicate that irradiation does not readily enhance corrosion kinetics, potentially due to severe lattice distortion and sluggish diffusion of metal atoms. Meanwhile, Wang et al. reported that structural defects induced by Xe^20+^ ion irradiation migrated and aggregated at the columnar grain boundaries of the Cr coating, accelerating the inward diffusion of oxygen and the outward diffusion of Zr and Cr, consequently affecting the coating’s service performance [7]. However, while amorphous MEA coatings are considered promising candidate materials for ATF coatings, the influence of irradiation on their corrosion behavior remains unclear. Therefore, it is imperative to investigate the corrosion mechanism and process of amorphous MEA coatings in high-temperature pressurized water, both with and without irradiation conditions [18].

In a prior study, two innovative amorphous MEA coatings (NbTiZr and Al_0.34_NbTiZr) were devised utilizing the magnetron sputtering technique and subjected to testing through Au ion irradiation. The findings revealed that the incorporation of Al elements resulted in markedly distinct irradiation responses between the two coatings [19,20]. The aim of this research was to ascertain how irradiation-induced structural alterations influence the corrosion performance of the coatings. In this context, we investigated the impact of ion irradiation on the corrosion resistance of two types of MEA-coated Zr substrates by comparing and analyzing the microstructural transformations and oxidation behaviors in identical corrosive environments between non-irradiated and irradiated specimens.

## 2. Materials and Methods

### 2.1. Material

Two types of amorphous NbTiZr and Al_0.34_NbTiZr MEA coatings were designed and fabricated on Zircaloy substrates using the magnetron co-sputtering technique. The substrates were sliced into small, thin specimens measuring 10 × 10 × 0.5 mm^3^ using low-speed wire electrical discharge machining. Prior to coating deposition, the surfaces of all substrate pieces were ground sequentially from 600 grit to 5000 grit using silicon carbide papers, followed by mechanical polishing with 0.5 μm diamond pastes to achieve a mirror-like finish. Subsequently, ultrasonic cleaning with alcohol was conducted for 30 min to eliminate surface contaminants and residual stress. High-purity sputtering composite targets, comprising four single-element metal targets of Nb, Ti, Zr, and Al, were employed. During the deposition process, the chamber vacuum pressure was maintained below 8 × 10^−4^ Pa, and the temperature was kept at approximately 300 °C to prevent phase transformation of the substrates. The flow rate of high-purity argon (Ar) was sustained at 51 sccm, and the chamber pressure was set at 0.53 Pa. The sputtering power and bias voltage for the prepared coatings were 200 W and −100 V, respectively. The coating thickness was controlled at approximately 5 μm, with a deposition time of around 2.5 h [19]. For convenience, the two MEA coatings are referred to as Al-0% and Al-10%, respectively. The nominal chemical compositions of the as-deposited coatings are presented in Table 1.

### 2.2. Au Ion Irradiation Experiment

**Figure 1:**
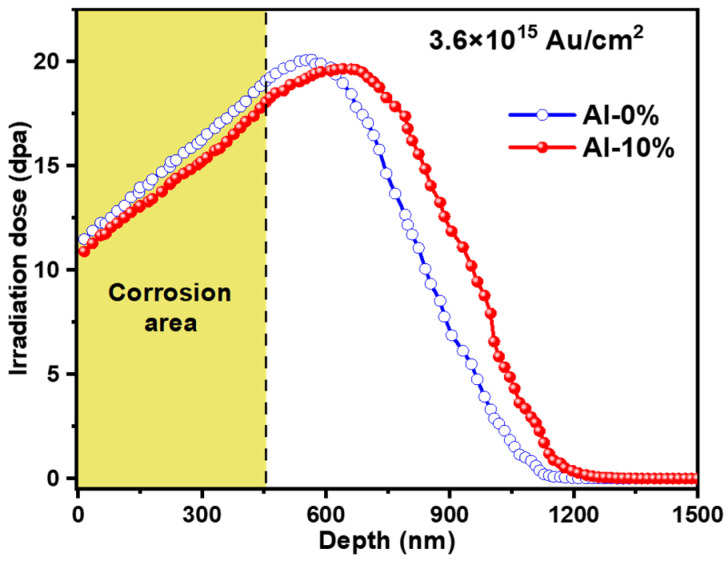
Irradiation damage doses as a function of depth in the Al-0% and Al-10% coatings irradiated with ions to 3.6 × 10^15^ Au/cm^2^ by SRIM simulation.

To simulate neutron irradiation, all irradiation experiments were conducted at room temperature using 6 MeV Au ions with a 2 × 3 MV tandem accelerator located at the Institute of Nuclear Science and Technology of Sichuan University. The vacuum pressure within the irradiation chamber was maintained at approximately 1 × 10^−4^ Pa. The Al-0% coating was irradiated with 6 MeV Au ions to a fluence of 3.6 × 10^15^ Au/cm^2^. For the Al-10% coating, the irradiation fluences were set at 1.8 × 10^15^ Au/cm^2^ and 3.6 × 10^15^ Au/cm^2^, respectively. The variation in the irradiation dose profile with respect to irradiation depth was calculated using the SRIM 2008 software with the quick Kinchin-Pease method [21]. For all coatings, the simulated maximum irradiation depth ranged from 1100 to 1200 nm, and the maximum corrosion area in this study is within the range indicated in Figure 1. As illustrated in Figure 1, the SRIM calculation results for the Al-0% and Al-10% coatings irradiated with a fluence of 3.6 × 10^15^ Au/cm^2^ are presented, with the corrosion region clearly marked.

### 2.3. Corrosion Experiment

The high-temperature pressurized water corrosion experiment was conducted in a custom-built, static autoclave constructed from Hastelloy alloy. To simulate the actual operating conditions of pressurized water reactors and investigate the impact of ion irradiation on corrosion behavior, all unirradiated and irradiated Al-0% and Al-10% coated specimens were subjected to deionized water containing 1000 ppm boron and a 0.01 mol/L LiOH solution for a duration of 30 days at 340 °C, with the corresponding saturation pressure at this temperature being 15.16 MPa. Two samples were prepared for each irradiation dose and for the as-deposited condition, resulting in a total of ten specimens placed in the autoclave. Following the corrosion experiment, all corroded samples were retrieved from the equipment and cleaned in deionized water for subsequent characterization.

### 2.4. Coating Characterization

The surface morphologies of both unirradiated and irradiated MEA coatings, before and after the corrosion experiment, were examined using a JEOL JSM-7500F field emission scanning electron microscope (SEM) (Akishima, Japan). The phase structures of the irradiated samples and corrosion oxidation products were analyzed via grazing incidence X-ray diffraction (GIXRD, SmartLab) (Kyoto, Japan), with the angle ranging from 20° to 90° at a scanning rate of 1°/min. The irradiation-induced structural changes in the irradiated coatings with varying fluences, as well as the microstructure of the thin oxide film, were characterized using a transmission electron microscope (TEM, Talos F200X, ThermoFisher) (Waltham, MA, USA) equipped with a Super-X energy dispersive spectroscopy detector (EDS) (Waltham, MA, USA). During TEM observation, selected area electron diffraction (SAED), scanning transmission electron microscopy (STEM) mode, high-resolution transmission electron microscopy (HRTEM), and corresponding fast Fourier transform (FFT) analyses were combined to determine the chemical composition and crystal structure of the corrosion products. The cross-sections of the irradiated samples and the oxide films on the corroded specimens were prepared using a focused ion beam (FIB) instrument with a Ga-ion beam.

## 3. Results

### 3.1. Microstructure Evolution After Au-Ion Irradiation

**Figure 2:**
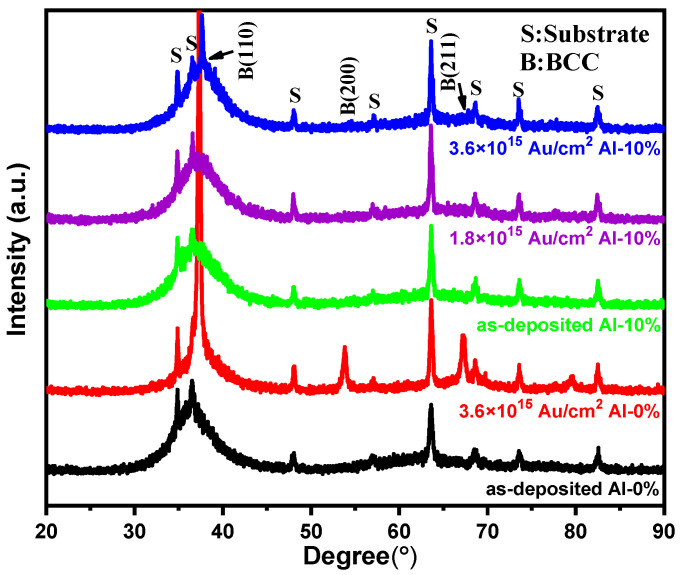
XRD spectra of the Al-0% and Al-10% coatings under non-irradiated conditions and at various ion fluences.

**Figure 3:**
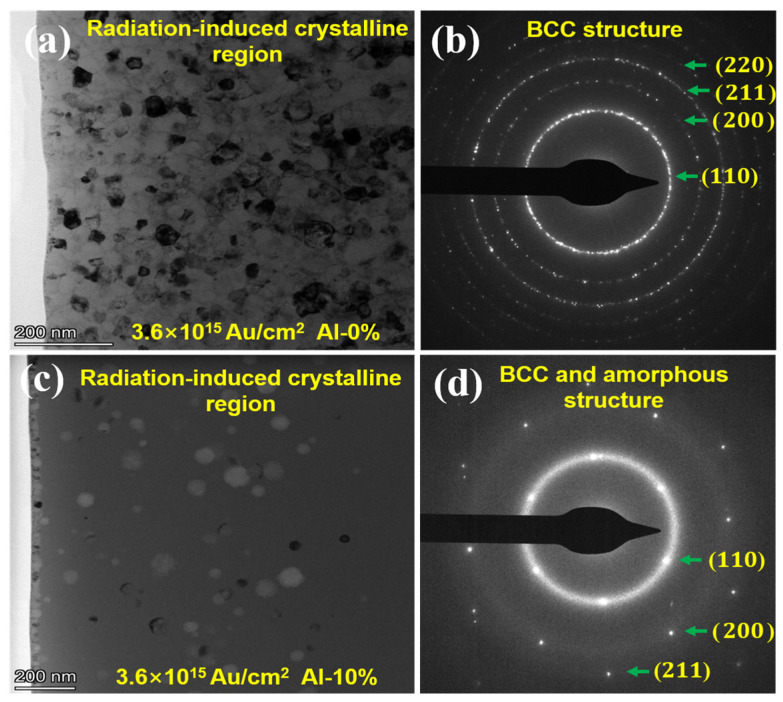
TEM observation of the cross-sectional microstructure of the coatings subjected to irradiation with a flux of 3.6 × 10^15^ Au/cm^2^: (**a**,**b**) the TEM-BF image and its corresponding SAED of the Al-0% coating; (**c**,**d**) TEM-BF image and its corresponding SAED of the Al-10% coating.

Figure 2 presents the XRD spectra of the Al-0% and Al-10% MEA coatings before and after irradiation under varying ion fluences. The XRD pattern of the irradiated Al-0% coating reveals numerous distinct sharp diffraction peaks, indexed to the (110), (200), (211), and (220) crystal planes, collectively indicative of a BCC crystalline structure. This finding suggests that crystallization occurred in the irradiated Al-0% coating. As depicted in Figure 2, the Al-10% coating subjected to an irradiation fluence of 1.8 × 10^15^ Au/cm^2^ exhibits broad diffraction peaks without any sharp peaks, consistent with the as-deposited state. When the irradiation fluence was increased to 3.6 × 10^15^ Au/cm^2^, a relatively sharp diffraction peak was observed at 2θ = 37.5°, corresponding to the (110) crystal plane of the BCC structure. This indicates a change in the phase structure of the Al-10% coating and the occurrence of irradiation-induced crystallization. For further analysis and characterization, typical cross-sectional TEM images illustrating the microstructural evolution of the Al-0% and Al-10% amorphous coatings after irradiation with a fluence of 3.6 × 10^15^ Au/cm^2^ are shown in Figure 3.

Figure 3a presents the bright-field TEM image of the Al-0% coating subjected to irradiation with a flux of 3.6 × 10^15^ Au/cm^2^. Nano-sized grains exhibiting varying contrasts are observed within the irradiated region, indicative of the phenomenon of irradiation-induced crystallization. From the surface towards the interior of the coating, both the size of the crystalline grains and the density of grain boundaries progressively increase with escalating irradiation dose. The SAED pattern depicted in Figure 3b for the irradiation-induced crystal region reveals four distinct diffraction rings, corresponding to the (110), (200), (211), and (220) crystal planes of the BCC structure. These findings demonstrate that high-dose irradiation can fully crystallize the irradiated area of the amorphous Al-0% coating, generating a substantial number of grain boundaries and irradiation-induced defects.

Similar characterization and analysis of the TEM observations of the cross-sectional microstructure were also conducted on the Al-10% coating, as depicted in Figure 3c,d. The bright-field TEM image of the Al-10% coating, subjected to irradiation with a fluence of 3.6 × 10^15^ Au/cm^2^, reveals numerous white nanocrystals of varying sizes aligned along the ion irradiation direction, along with a continuous layer of crystals near the coating surface. This observation differs from that of the irradiated Al-0% sample. The SAED pattern displays distinct sharp diffraction spots corresponding to the (110), (200), and (211) crystal planes of the body-centered cubic structure, in addition to a prominent amorphous diffraction ring. The results indicate that the amorphous Al-0% coating underwent partial crystallization upon irradiation, generating a substantial number of grain boundaries and defects. It is noteworthy that prior studies have reported on the effects of ion irradiation on the microstructure of Al-0% and Al-10% amorphous coatings, with detailed reports available in reference [20].

### 3.2. Surface Morphologies and XRD Analysis of Oxide Films

**Figure 4:**
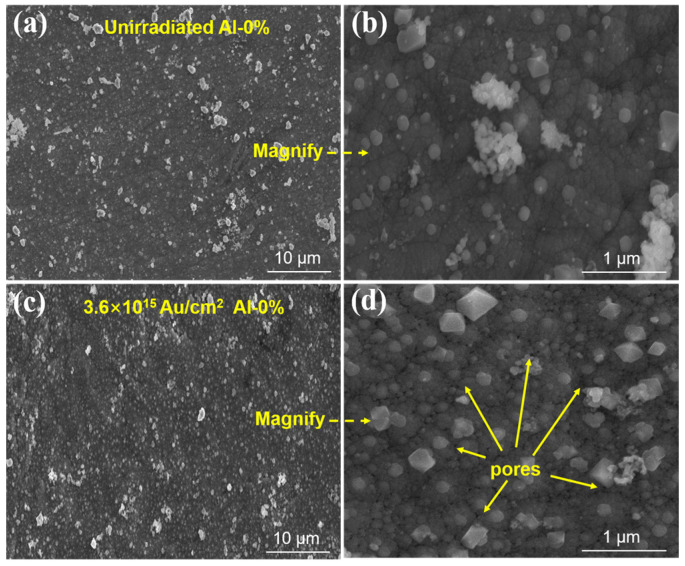
SEM observation of the surface morphology of oxide films formed on unirradiated and irradiated Al-0% coatings after corrosion: (**a**,**b**) unirradiated sample; (**c**,**d**) irradiated sample.

After exposure to the high-temperature pressurized water corrosive environment at 340 °C for 30 days, the surface morphologies of the unirradiated and irradiated Al-0% MEA coatings were characterized by using SEM, as shown in Figure 4a–d. As can be shown from Figure 4a,b, the isolated irregular-shaped oxide particles were randomly distributed on the surface of the unirradiated Al-0% coating, and the magnified image of Figure 4a exhibited some small round and hexagonal oxide particles and clusters composed of agminated oxide particles. Figure 4c shows that the particle size for the irradiated coating was relatively small compared to the unirradiated one, while the small oxide-particle area density seems to be higher for the unirradiated coating. Meanwhile, as shown in Figure 4d, it is noted that fine circular oxides were mainly distributed on the grain boundary of the inner oxide layer, and tiny pores can be observed on the grain boundary, which would dramatically degenerate protection effect.

**Figure 5:**
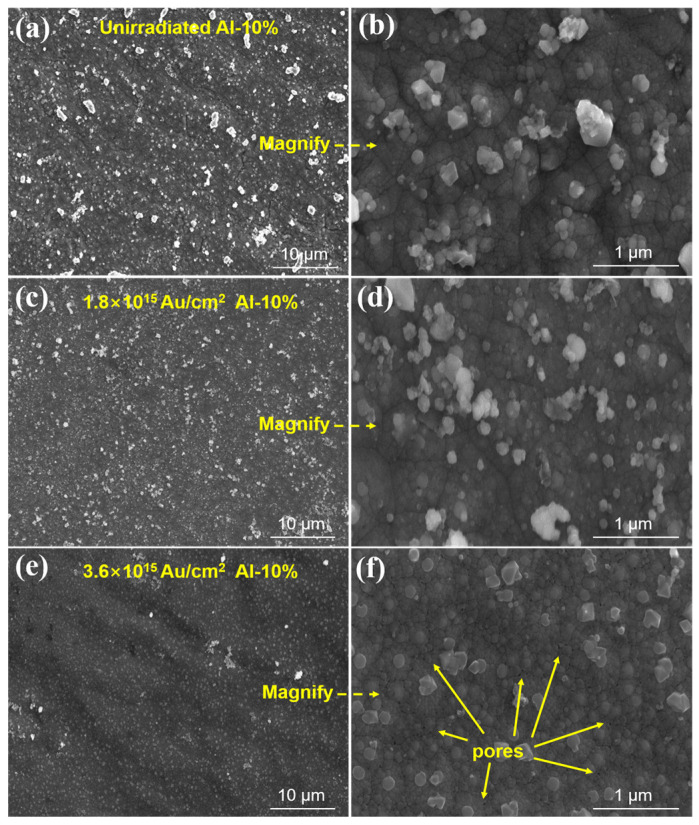
SEM observation of the surface morphology of oxide films formed on unirradiated and irradiated Al-10% coatings after corrosion: (**a**,**b**) unirradiated sample; (**c**,**d**) 1.8 × 10^15^ Au/cm^2^ irradiated sample; (**e**,**f**) 3.6 × 10^15^ Au/cm2 irradiated sample.

Figure 5a–f present the surface morphologies of the Al-10% amorphous MEA coatings irradiated with various fluences and corroded for 30 days. For the Al-10% MEA coating, the size of oxide particles and the number of oxide clusters decreased with the increase in irradiation fluences, which was mainly because ion irradiation promotes the viscous flow on the coating surface, makes collective atoms participate in the displacement and rearrangement, leads to the homogenization of the amorphous phase, reduces the nucleation point of oxides and inhibits their growth. In addition, tiny corrosion holes appeared on the surface of the high-fluence irradiated specimen following corrosion, while the low-fluence irradiated one did not show any [22].

**Figure 6:**
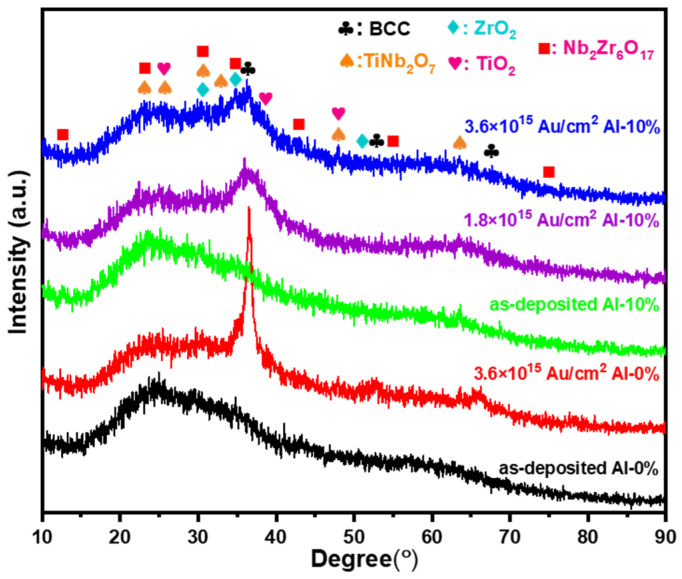
GIXRD spectra obtained from both non-irradiated and irradiated Al-0% and Al-10% coatings after high temperature and high pressure water corrosion.

Figure 6 shows the GIXRD spectra of the irradiated Al-0% and Al-10% coatings and non-irradiated specimens after exposure to the high-temperature pressurized water environment at 340 °C for 30 days. According to the GIXRD diffractograms, the types of corrosion products of all the coatings were almost identical, and the diffraction peaks corresponding to orthorhombic Nb_2_Zr_6_O_17_ (PDF No.09-0251), monoclinic TiNb_2_O_7_ (PDF No.72-0116), tetragonal TiO_2_ (PDF No.84-1285) and cubic ZrO_2_ (PDF No.49-1642) were observed, indicating that the coatings surface were covered by the above oxide products. In order to further determine the oxidation products of each coating after corrosion, TEM would be used for further characterization [23].

### 3.3. Cross-Sectional Microstructure of the Oxide Film of Al-0% Coating

#### 3.3.1. Oxide Film Formed on Unirradiated Coating

**Figure 7:**
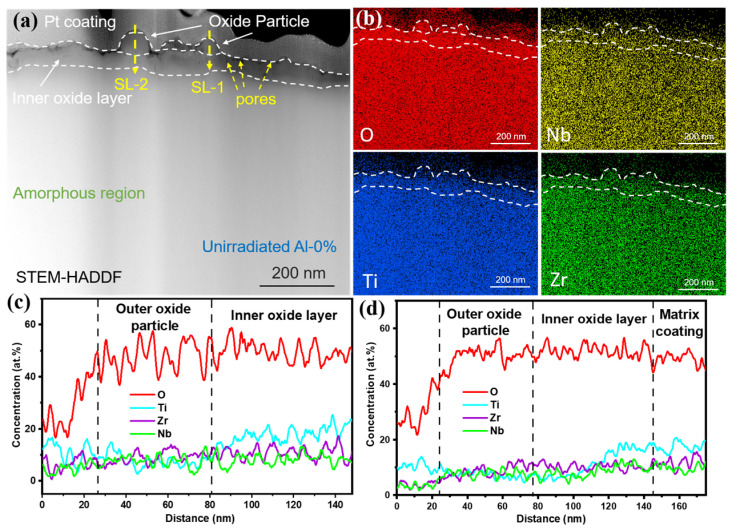
Compositional analysis of the oxide film formed on the unirradiated Al-0% coating following 30-day exposure: (**a**) cross-sectional STEM-HAADF image; (**b**) EDS maps of O, Nb, Ti and Zr, respectively; (**c**,**d**) composition profiles corresponding to the scanning lines (SL-1 and SL-2).

Figure 7 exhibits the cross-sectional TEM morphology observation and EDS results of the oxide film formed on the as-deposited Al-0% coupon corroded for 30 days. The STEM-HAADF image and corresponding element mappings indicate that the oxide scale has a typical double-layer structure, consisting of dispersed external oxide particles and a continuous and loose inner oxide layer beneath these particles (Figure 7a). The thickness of the inner oxide layer ranges from 62 to 102 nm, with an average of 84 nm, and some small corrosion holes in the oxide film were also observed. According to the chemical composition profile from EDS line scanning and mapping images, both the outer oxide particles and the inner oxide layer are mainly composed of Nb, Ti and Zr oxides, but the composition is slightly different (Figure 7b–d).

**Figure 8:**
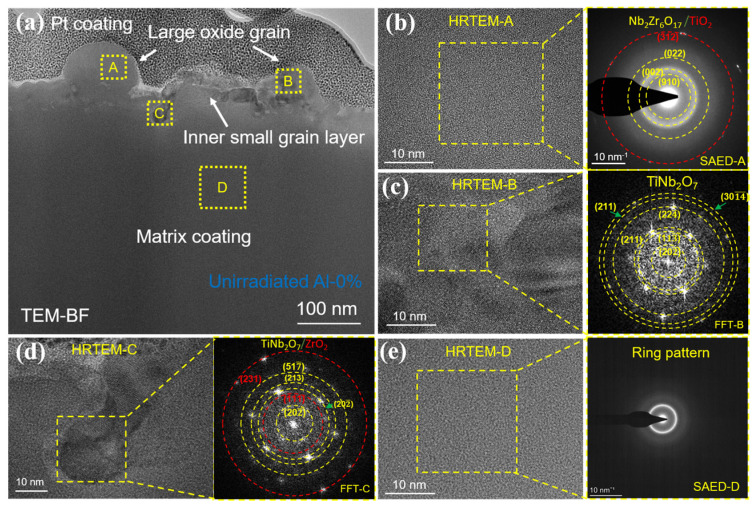
Microstructure of the oxide film formed on the unirradiated Al-0% coating following 30-day exposure: (**a**) TEM-BF image showing a bilayer structure of the oxide film; (**b**) HRTEM image and the corresponding SAED pattern of region A; (**c**) HRTEM image and the corresponding FFT pattern of region B; (**d**) HRTEM image and the corresponding FFT pattern of region C; (**e**) HRTEM image and SAED pattern of region D.

The TEM-BF image of the oxide scale of the Al-0% coating is observed in Figure 8a; a dual-layer structure can be shown derived from the different microscopic morphologies and oxide products. The outer layer consists of large dispersed oxide particles with a size of about a hundred nanometers, and the inner oxide layer is composed of small grains. From the HRTEM image and corresponding SAED pattern of the outermost oxide particle of region A in Figure 8b, it can be shown that the diffraction pattern corresponds to oxide products are crystalline TiO_2_ and amorphous Nb_2_Zr_6_O_17_, which is in line with the GIXRD result in Figure 6. In addition, according to Figure 8c, the HRTEM picture as well as the FFT image of region B suggest that the TiNb_2_O_7_ oxide particle grew on the specimen surface. From the HRTEM and corresponding FFT pattern of region C (Figure 8d), small TiNb_2_O_7_ oxides were embedded in the ZrO_2_-based internal oxide layer, in accordance with the GIXRD pattern. From the HRTEM and SAED image of region D (Figure 8e), the microstructure of the uncorroded region of the coating remains in an amorphous state and exhibits the typical diffraction ring. The interface near the inner oxide layer and residual coating is relatively dense, and there are no visible holes and cracks.

#### 3.3.2. Oxide Film Formed on Irradiated Coating

**Figure 9:**
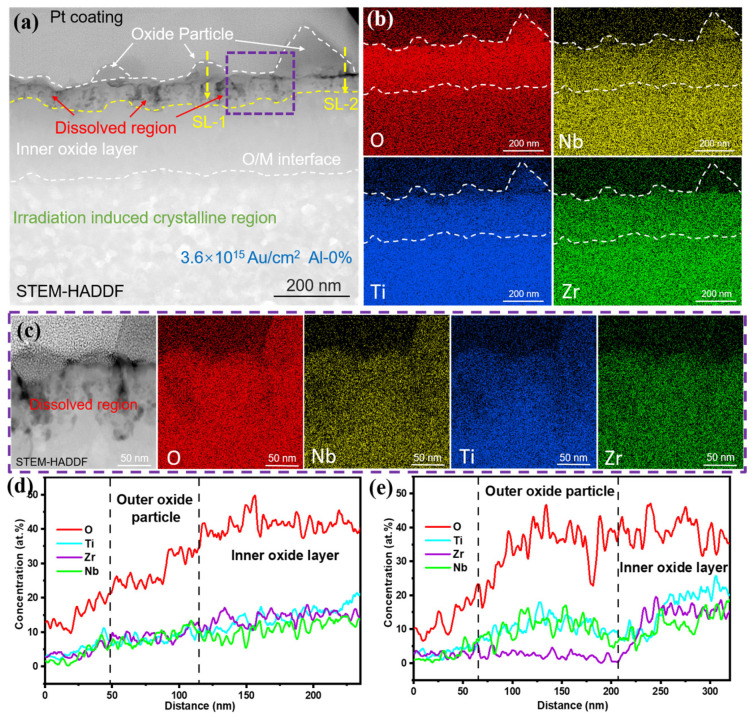
Compositional analysis of the oxide film formed on the irradiated Al-0% coating following 30-day exposure: (**a**) cross-sectional STEM-HAADF image; (**b**) EDS maps of O, Nb, Ti and Zr, respectively; (**c**) STEM-HAADF image and EDS mapping of the dissolved region marked in (**a**) by a purple rectangle; (**d**,**e**) composition profiles corresponding to the scanning lines (SL-1 and SL-2).

Figure 9 shows the STEM-HAADF image and the cross-sectional composition analysis of the oxide scale formed on the irradiated specimen with a fluence of 3.6 × 10^15^ Au/cm^2^ following corrosion for 30 days. As shown in Figure 9a, the double-layer structure of the oxide film was formed on the Al-0% coating corroded after irradiation. From Figure 9b–e, the oxide particles of different sizes were distributed in the outer layer, and the thickness of the inner oxide layer varies over a range of 280–330 nm, with an average of 305 nm, which is much larger than the unirradiated sample (Figure 7). The upper part of the internal oxide layer, that is, the surface in direct contact with the oxidant, had a dissolution region with a width in the range of 50–130 nm, and there were small corrosion holes embedded in it. In the lower part of the inner oxide layer, grains with different contrast in the irradiation-induced crystallization region can still be observed. Meanwhile, according to Figure 9a, no corrosion-induced defects such as cracks and pores were observed at the interface between the oxidation zone and the unoxidized matrix coating. However, no obvious grains were found in the internal oxide layer below the dissolution region, indicating that corrosion led to the disappearance of the crystalline region originally induced by irradiation. According to element mapping images (Figure 9c), in addition, the chemical contents of Nb, Ti and Zr in the dissolved region were lower than those of other oxidation areas, especially the depletion of the Ti element was the most obvious.

**Figure 10:**
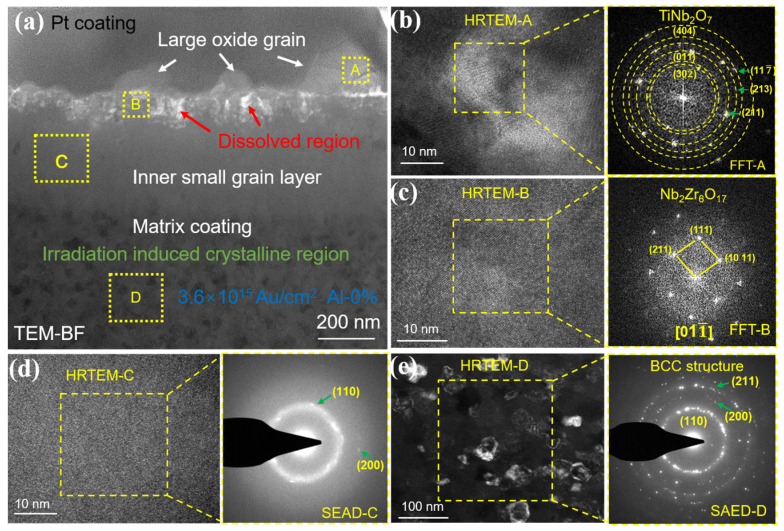
Microstructure of the oxide film formed on the irradiated Al-0% coating following 30-day exposure: (**a**) TEM-BF image showing a bilayer structure of the oxide film; (**b**) HRTEM image and the corresponding FFT pattern of region A; (**c**) HRTEM image and the corresponding FFT pattern of region B; (**d**) HRTEM image and the corresponding SAED pattern of region C; (**e**) HRTEM image and SAED pattern of region D.

The bright-field image of the oxide film grown on the irradiated Al-0% coating exposed to a corrosion environment is shown in Figure 10a, indicating the different contrast between the exterior oxide particles, the dissolved region, the interior oxidation layer and the irradiation-induced crystalline zone, which is consistent with the results shown in the HAADF-STEM and EDS images. Combining the element scanning diagrams (Figure 9b) with HRTEM pictures and FFT patterns (Figure 10b), it can be seen that the oxide particle of region A was a Nb- and Ti-rich oxide, which was labeled as monoclinic TiNb_2_O_7_. In region B of the dissolution zone (Figure 10c), the orthorhombic Nb_2_Zr_6_O_17_ phase can be indexed from its HRTEM and FFT pattern, similar to the unirradiated one. From the HRTEM and SAED images of region C (Figure 10d), the appearance of amorphous diffraction rings can be clearly observed, accompanied by some diffraction lattice spots of (110) and (200) crystal planes, which was in accordance with the regions with different contrast shown in the HAADF-STEM picture (Figure 9a). The region D below the intermediate oxide layer remains in the previous state, i.e., the irradiation-induced crystallization area, indicating that this area was not completely affected by high-temperature pressurized corrosion (Figure 10e). Based on the above results, it can be clearly seen that irradiation accelerates the corrosion kinetics of the Al-0% coating.

### 3.4. Cross-Sectional Microstructure of the Oxide Film of Al-10% Coating

#### 3.4.1. Oxide Film Formed on Unirradiated Coating

**Figure 11:**
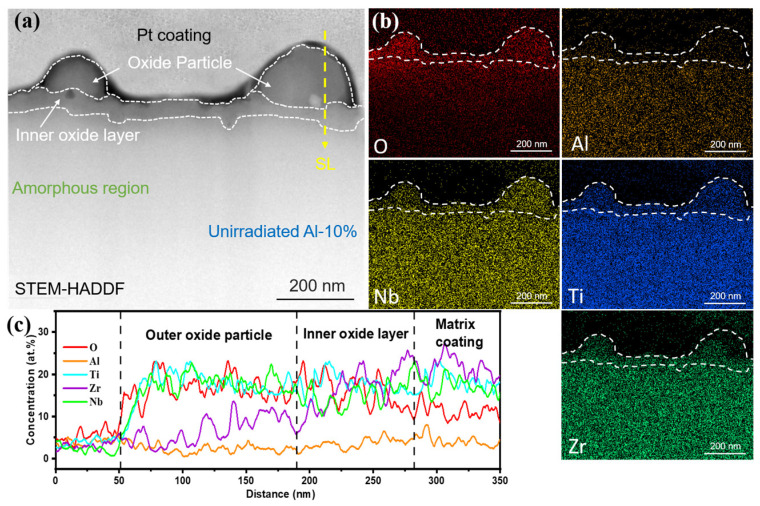
Compositional analysis of the oxide film formed on the unirradiated Al-10% coating following 30-day exposure: (**a**) cross-sectional STEM-HAADF image; (**b**) EDS maps of O, Al, Nb, Ti and Zr, respectively; (**c**) composition profiles corresponding to the scanning lines (SL-1).

Figure 11 shows the STEM-HAADF and EDS image of the oxide layer after the corrosion of the Al-10% coating without irradiation. Similar to the un-irradiated Al-0% coating, the oxide region is composed of two layers, and some corrosion holes can be observed between the inner and outer oxide layers (Figure 11a). The outer oxide particles and the inner oxide layer are rich in Nb, Ti and Zr elements (Figure 11b,c). It is worth mentioning that there is no obvious enrichment of Al in the oxidation area, which may be caused by the dissolution of Al in the corrosive medium, as has been reported before. The oxide particles with hundreds of nanometers are distributed in the outer layer, while the thickness of the inner oxide layer fluctuates in the range of 40~90 nm, with an average of 65 nm, which is smaller than the un-irradiated Al-free coating.

**Figure 12:**
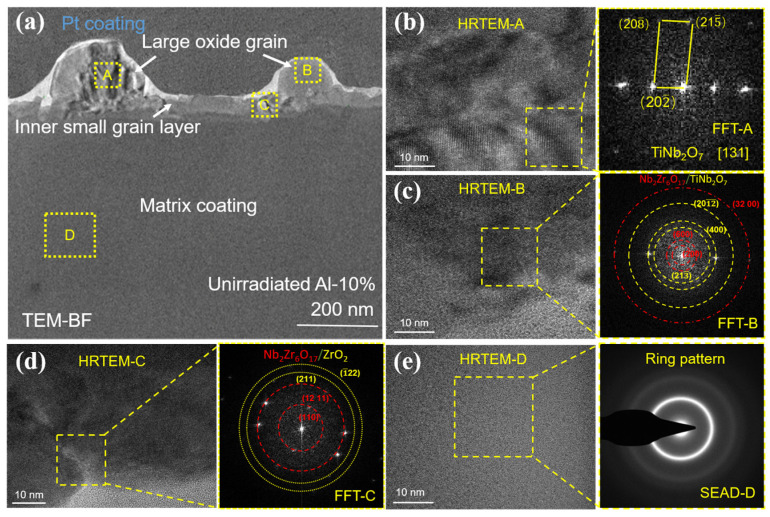
Microstructure of the oxide film formed on the unirradiated Al-10% coating following 30-day exposure: (**a**) TEM-BF image showing a bilayer structure of the oxide film; (**b**) HRTEM image and the corresponding FFT pattern of region A; (**c**) HRTEM image and the corresponding FFT pattern of region B; (**d**) HRTEM image and the corresponding FFT pattern of region C; (**e**) HRTEM image and SAED pattern of region D.

Figure 12 shows the TEM characterization of the cross-section of the oxide layer in the non-irradiated area of the Al-10% coating after high-temperature water corrosion. Figure 12a shows a cross-sectional tem-bf image of the entire region of interest, where regions A, B and C correspond to external oxide particles and internal oxide layers, respectively, and region D is an uncorroded coating. The HRTEM and FFT images of region A (Figure 12b) show that the oxide is a monoclinic TiNb_2_O_7_ phase (PDF No.72-0116). In region B (Figure 12c), the mixed oxide containing orthorhombic Nb_2_Zr_6_O_17_ (PDF No.09-0251) and monoclinic TiNb_2_O_7_ phase can also be analyzed from its HRTEM image and FFT diagram, while the analysis results in region C (Figure 12d) show that the internal oxide layer is mainly composed of Nb_2_Zr_6_O_17_ and ZrO_2_ (PDF No.49-1642). The non-corroded area D (Figure 12e) of the coating still retains the original amorphous structure. Compared with the non-irradiated Al-0% coating, the penetration depth of oxygen in the deposited Al-10% coating containing a small amount of Al element after high-temperature water corrosion is reduced, which corresponds to the SEM characterization results after corrosion in the previous chapter.

#### 3.4.2. Oxide Film Formed on Irradiated Coating with a Fluence of 1.8 × 10^15^ Au/cm^2^

**Figure 13:**
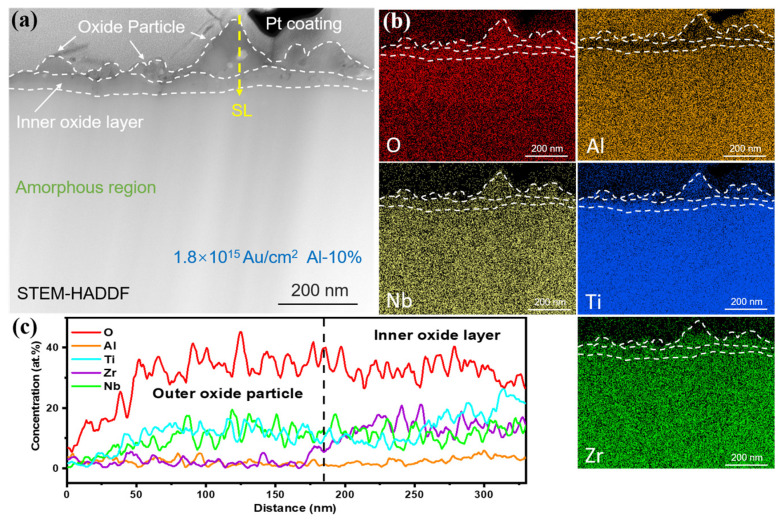
Compositional analysis of the oxide film formed on the irradiated (1.8 × 10^15^ Au/cm^2^) Al-10% coating following 30-day exposure: (**a**) cross-sectional STEM-HAADF image; (**b**) EDS maps of O, Al, Nb, Ti and Zr, respectively; (**c**) composition profiles corresponding to the scanning lines (SL-1).

**Figure 14:**
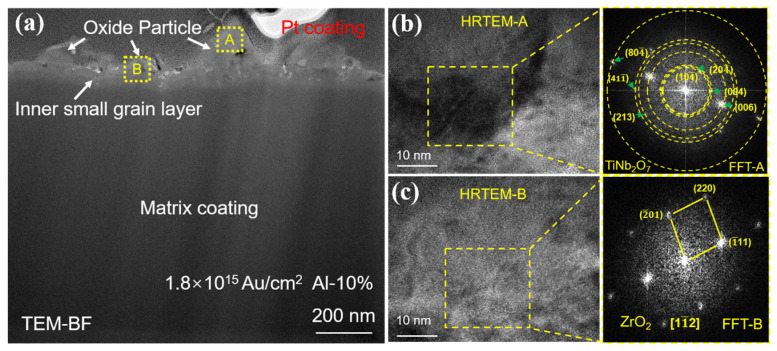
Microstructure of the oxide film formed on the irradiated (1.8 × 10^15^ Au/cm^2^) Al-10% coating following 30-day exposure: (**a**) TEM-BF image showing a bilayer structure of the oxide film; (**b**) HRTEM image and the corresponding FFT pattern of region A; (**c**) HRTEM image and the corresponding FFT pattern of region B.

The chemical composition and microstructure of the oxide film grown on the irradiated Al-10% coating, subjected to a fluence of 1.8 × 10^15^ Au/cm^2^, were analyzed using TEM following corrosion. Figure 13 and Figure 14, respectively, illustrate the compositional distribution and TEM phase characterization of the oxide layer after corrosion of the irradiated Al-10% coating. The HAADF-STEM image reveals the formation of oxide particles of varying sizes on the coating surface post-corrosion, along with the presence of some corrosion pores between the external oxide particles and the internal oxide layer (Figure 13a). The thickness of the inner oxide layer ranges from 40 to 94 nm, with an average value of 67 nm, which is essentially consistent with that of the unirradiated coating. The elemental surface distribution results indicate that the outer oxide particles are enriched with Nb and Ti elements, whereas the inner layer is predominantly composed of Zr elements (Figure 13b,c). Further examination of the oxide film microstructure is presented in Figure 14. In the cross-sectional TEM-BF pictures, the two-layer structure of the oxide film remains distinctly visible (Figure 14a). Through analysis and calibration of the HRTEM images and corresponding FFT images of regions A and B (Figure 14b,c), it can be concluded that the internal and external oxide layers are mainly composed of ZrO_2_ and TiNb_2_O_7_, respectively.

#### 3.4.3. Oxide Film Formed on Irradiated Coating with a Fluence of 3.6 × 10^15^ Au/cm^2^

**Figure 15:**
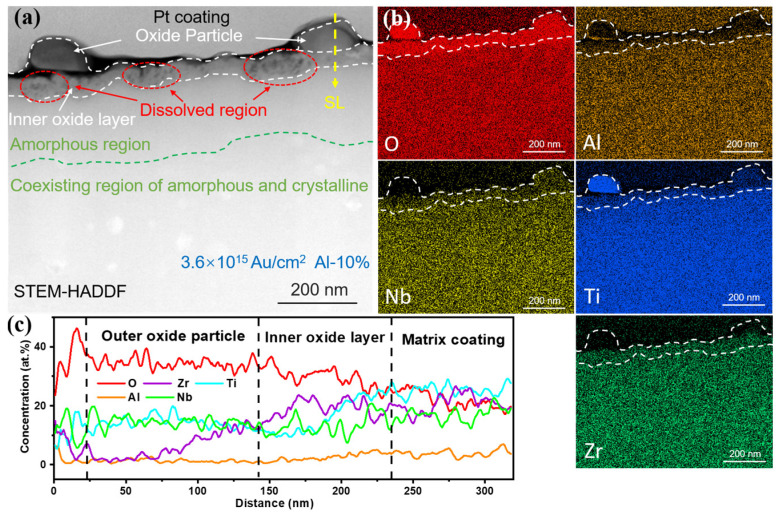
Compositional analysis of the oxide film formed on the irradiated (3.6 × 10^15^ Au/cm^2^) Al-10% coating following 30-day exposure: (**a**) cross-sectional STEM-HAADF image; (**b**) EDS maps of O, Al, Nb, Ti and Zr, respectively; (**c**) composition profiles corresponding to the scanning lines (SL-1).

When the irradiation fluence was increased to 3.6 × 10^15^ Au/cm^2^, the corrosion behavior of the Al-10% coating also became more severe. The STEM-HAADF image in Figure 15 revealed the presence of scattered large oxide particles and distinct corrosion dissolution regions on the coating surface, which closely resembled the corrosion observed in of the Al-0% coating sample after high-fluence irradiation (Figure 15a). However, the corrosion degree of corrosion in the dissolution regions was lower than that in the Al-0% coating. The thickness of the internal oxide film fluctuated within the range of 45–105 nm, with an average measured value of 75 nm (Figure 15b,c), which was greater than the thickness of the internal oxide film in the Al-10% coating irradiated at a fluence of 1.8 × 10^15^ Au/cm^2^. The outer oxide particles predominantly consisted of Ti-rich oxides, while the inner oxide layer was composed of oxides rich in Nb-, Ti-, and Zr-rich oxides.

**Figure 16:**
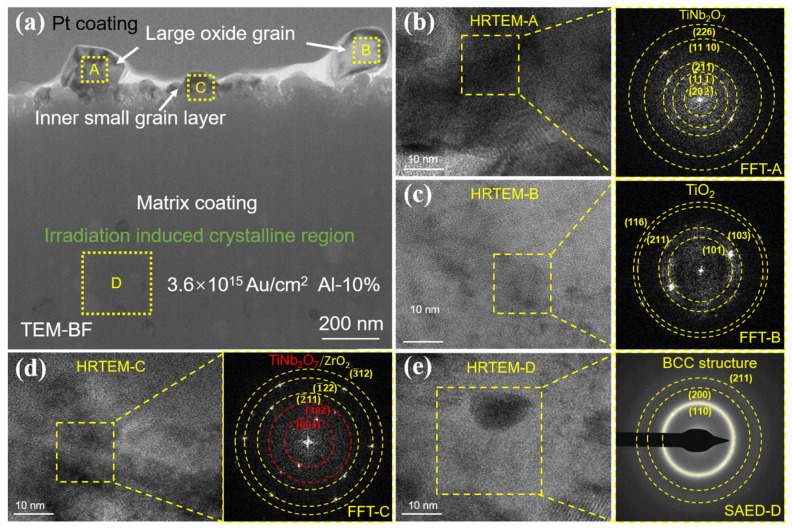
Microstructure of the oxide film formed on the irradiated (3.6 × 10^15^ Au/cm^2^) Al-10% coating following 30-day exposure: (**a**) TEM-BF image showing a bilayer structure of the oxide film; (**b**) HRTEM image and the corresponding FFT pattern of region A; (**c**) HRTEM image and the corresponding FFT pattern of region B; (**d**) HRTEM image and the corresponding FFT pattern of region C; (**e**) HRTEM image and the corresponding SAED pattern of region D.

Further analysis of the microstructure of the Al-10% coating oxide film, as depicted in Figure 16, showed that the cross-sectional TEM-BF images can still clearly distinguish the oxide film as a two-layer structure (Figure 16a). By analyzing and calibrating the HRTEM images and corresponding FFT images of regions A, B, and C (Figure 16b–d), it was determined that the external oxide layer was primarily composed of TiNb_2_O_7_ and TiO_2_, whereas the internal oxide layer mainly consisted of a mixed oxide of TiNb_2_O_7_ and ZrO_2_. Region D (Figure 16e) represented an unoxidized coating, with its structure remaining in its original state.

### 3.5. Summary of Key Experimental Observations

**Figure 17:**
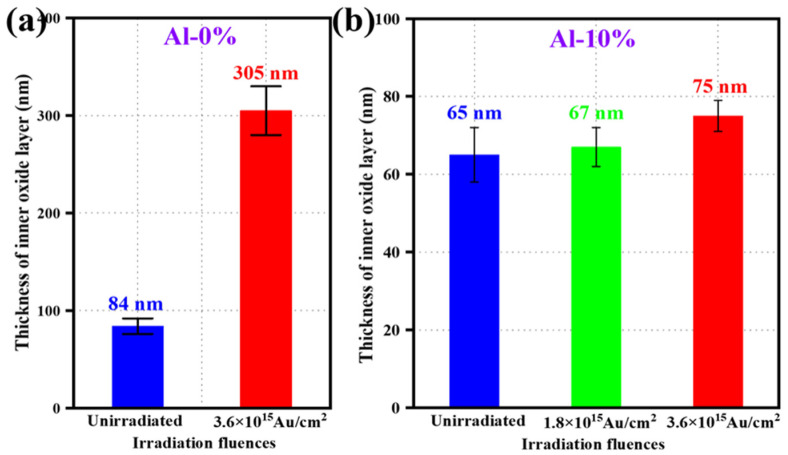
Thickness of the internal oxide layer in the unirradiated and irradiated regions of the coating after corrosion: (**a**) Al-0%; (**b**) Al-10%.

A comprehensive comparison demonstrates that with an increase in ion irradiation dose, the corrosion severity of the Al-10% coating escalates, and the corrosion kinetics are expedited. When contrasting Al-0% and Al-10% coatings subjected to the same irradiation dose (3.6 × 10^15^ Au/cm^2^), the latter displays markedly superior resistance to radiation-induced corrosion. Furthermore, it is noteworthy that, irrespective of irradiation, the oxide films formed on all coatings post-corrosion exhibit a bilayer structure, comprising an outer layer rich in Nb and Ti oxides and an inner layer abundant in Zr oxides. The internal oxide layer in irradiated regions is consistently thicker compared to that in non-irradiated regions. As depicted in Figure 17, the thickness of the internal oxide layer in the non-irradiated area of the Al-0% coating measures 84 ± 14 nm, whereas in the irradiated area, it is 305 ± 25 nm. For the Al-10% coating, the internal oxide layer thickness in the non-irradiated area is 65 ± 7 nm, while following irradiation at doses of 1.8 × 10^15^ Au/cm^2^ and 3.6 × 10^15^ Au/cm^2^, the respective thicknesses are 67 ± 5 nm and 75 ± 4 nm. There was no significant difference in the thickness of the inner oxide layer between the low-fluence irradiated and non-irradiated coatings. These results indicate that high-fluence irradiation accelerates the rate of high-temperature water corrosion in the AlNbTiZr coatings.

## 4. Discussion

**Figure 18:**
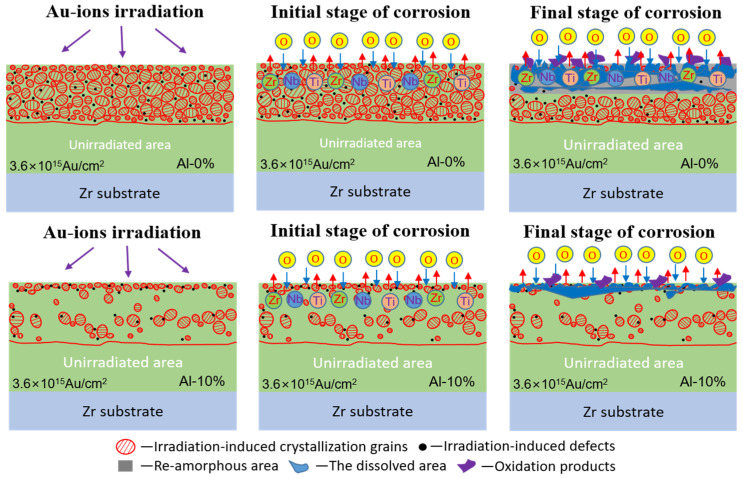
Schematic illustration of the corrosion process for AI-0% and Al-10% amorphous coatings post-irradiation.

Based on the aforementioned series of studies, we have gained a comprehensive understanding of the high-temperature water corrosion process of AlNbTiZr coating samples both before and after gold ion irradiation. Building upon these research findings, we have conducted a meticulous analysis of the high-temperature water corrosion process of Al-0% and Al-10% coatings, both pre- and post-ion irradiation, to enhance our comprehension of the corrosion mechanism. The corrosion processes of the aforementioned AI-0% and Al-10% amorphous coatings following irradiation are depicted in Figure 18.

### 4.1. Formation Mechanism of Oxides in Coatings

Both the oxide films of unirradiated and irradiated regions of the two coatings exhibit a bilayer structure, comprising an outer layer rich in Nb and Ti oxide particles and an inner layer rich in Zr oxide. These oxides are formed through the reaction between the alloying elements and oxygen in the high-temperature aqueous environment. The primary oxidation reactions of the alloying elements with high-temperature water can be expressed as follows:

2Al + 3H_2_O → Al_2_O_3_ + 3H_2_↑

Al + H_2_O → AlOOH + H_2_↑

Zr + 2H_2_O → ZrO_2_ + 2H_2_↑

Ti + 2H_2_O → TiO_2_ + 2H_2_↑

2Nb + 5H_2_O → Nb_2_O_5_ + 5H_2_↑

During prolonged exposure, complex oxides form via solid-state reactions among the initially generated oxides:

TiO_2_ + Nb_2_O_5_ → TiNb_2_O_7_

Nb_2_O_5_ + 6ZrO_2_ → Nb_2_Zr_6_O_17_

The outer oxide granular layer forms through nucleation and growth at the coating/water interface, whereas the inner oxide layer develops via the inward diffusion of oxygen to the interface between the outer oxide and the uncorroded coating. Notably, the growth space for the inner layer is furnished by surface pores and cross-sectional dissolution regions, both created by vacancy accumulation resulting from the outward diffusion of alloying elements. These porous networks serve as facile pathways for oxygen ingress, further facilitating internal oxidation [24,25].

The likelihood of oxide formation is dictated by thermodynamic selectivity. According to the oxygen potential diagram [26], the standard Gibbs free energies of formation (∆*_f_G*°) follow the sequence: Al_2_O_3_ > ZrO_2_ > TiO_2_ > Nb_2_O_5_. Although this hierarchy suggests Al has the highest affinity for oxygen, no Al-containing oxides were detected in the scale. This phenomenon can be attributed to three factors: (1) The relatively low Al content and insufficient experimental temperature to crystallize stable Al_2_O_3_; (2) The formation of soluble AlOOH in high-temperature water, which detaches rapidly, depleting Al from the coating [27]; (3) The preferential occupation of oxygen adsorption sites by Ti and Zr atoms, coupled with the weakening of O–Al bond strength by Ti, thereby reducing the stability of Al-containing phases [28]. The growth of the oxide film is governed by the oxygen potential gradient within the high-temperature, high-pressure medium. During the initial corrosion stage, metastable or amorphous precursors of Ti, Zr, and Nb form via the diffusion and migration of metal cations and oxygen anions. These precursors subsequently undergo thermal activation and crystallize into stable single oxides (TiO_2_, ZrO_2_, Nb_2_O_5_). Upon prolonged exposure, these single oxides react hydrothermally to form thermodynamically more stable composite oxides, namely TiNb_2_O_7_ and Nb_2_Zr_6_O_17_. These composite phases propagate along pores, micro-cracks, and dissolution regions within the coating, constituting the stable oxidation products in the final stages of corrosion (Figure 18).

In summary, the corrosion evolution of the AlNbTiZr coating is a synergistic process controlled by both thermodynamics and kinetics. The ∆*_f_G*° hierarchy establishes the driving force for oxidation, while the outward diffusion of cations and the resultant porous architecture dictate the mass transport of oxygen. Despite the high thermodynamic affinity of Al, its loss via dissolution and site competition leads to the dominance of Zr- and Ti-based oxides. Consequently, the observed bilayer structure—an outer granular layer enriched in Ti/Nb oxides and an inner layer dominated by Zr-based oxides—represents the final equilibrium state governed by the interplay of these factors, which ultimately determines the long-term corrosion resistance of the coating.

### 4.2. Irradiation Effect on the Corrosion Properties of Coatings

Figure 18 clearly demonstrates that high-fluence ion irradiation significantly accelerates the high-temperature, high-pressure water corrosion of the coatings, as evidenced by the substantially thicker oxide scales in the irradiated regions compared to their non-irradiated counterparts. This discrepancy primarily stems from irradiation-induced crystallization and the resultant acceleration of elemental diffusion.

Following high-dose irradiation, the Al-0% coating exhibited extensive crystallization, characterized by finer grains with a higher grain boundary density near the surface, while larger grains with a reduced boundary density dominated the peak damage region. Similarly, a ~40 nm crystalline layer formed on the surface of the Al-10% coating, displaying a gradient grain size distribution. Notably, in the Al-10% coating, these crystalline grains were sporadically distributed within the residual amorphous matrix. The underlying mechanism involves irradiation-induced super-saturation of point defects and deposited energy, which enhance atomic mobility and facilitate diffusional rearrangement. Consequently, numerous grain boundaries and pre-existing irradiation-induced defects (e.g., dislocation loops and cascades) were introduced, acting as short-circuit diffusion pathways that accelerated both cation out-diffusion and oxygen ingress, thereby leading to distinct surface dissolution zones.

A striking observation is the partial re-amorphization of these initially crystallized regions within the Al-0% coating after corrosion. This phenomenon represents a classic case of environment-induced stability inversion in multi-principal-element alloys, governed by a synergy of thermodynamic and kinetic factors. Thermodynamically, at the nanoscale, the total Gibbs free energy is dominated by interface energy. The ultrafine crystalline grains possess intrinsically high surface energies; thus, transitioning to an amorphous phase—which offers superior structural flexibility to relax interfacial stresses—results in a lower overall free energy compared to the fragmented nano-crystalline state. Kinetically, the preferential leaching of highly reactive elements (Al, Ti, Zr and Nb) shifts the local chemistry toward a range with higher glass-forming ability (GFA). Concurrently, the irradiation-corrosion synergistic disordering process exacerbates lattice instability; the pre-existing defects and the insufficient oxygen supply within the confined corrosion environment collectively lower the kinetic barrier for structural relaxation into a disordered state, facilitating the formation of a metastable amorphous oxide layer [29,30].

Compared to the coating without Al, the radiation resistance of the A-10% coating exhibits a marked enhancement; the incorporation of Al significantly bolsters the coating’s resistance to radiation-induced phase transformation. This improvement is primarily attributed to the introduction of Al with a smaller atomic radius, which reduces the free volume, thereby increasing viscosity and slowing down diffusion, and improving the mixing entropy within the coating system [31,32,33,34].

## 5. Conclusions

The corrosion behaviors in a simulated PWR water environment were investigated on two MEA coatings before and after Au ion irradiated conditions. The following conclusions can be drawn from this investigation:

(1) The high-temperature and high-pressure water corrosion tests conducted on the two MEA coatings before and after irradiation revealed the formation of double-layer oxide films, composed of oxides of Zr, Ti, and Nb.

(2) Upon corrosion of the irradiated coating, distinct dissolution zones and micro-pores emerged on its surface. High-dose irradiation induced microstructural alterations and radiation damage, which accelerated the corrosion rate of the coating and significantly enhanced its corrosion kinetics.

(3) Upon the addition of a small amount of Al, the radiation resistance of the coating significantly improves, and its resistance to radiation-induced phase transitions is markedly enhanced. Concurrently, the incorporation of Al stabilizes the amorphous structure of the coating, leading to a substantial reduction in the irradiation-accelerated corrosion effect. This enhancement is primarily attributed to the slowing down of the diffusion effect and the increased mixing entropy within the coating system.

## Figures and Tables

**Table 1:** The nominal compositions (at.%) of the as-spun Al-0% and Al-10% coatings.

MEA Coating	Al	Nb	Ti	Zr
Al-0%	/	32.4	33.5	34.1
Al-10%	10.2	29.7	29.4	30.7

## Data Availability

The data presented in this study are available on request from the corresponding author. The data are not publicly available due to privacy concerns.
